# Glymphatic System Dysfunction in Central Nervous System Diseases

**DOI:** 10.1002/cns.70810

**Published:** 2026-03-06

**Authors:** Anwar Zahran, Omar Abu‐Khazneh, Mohammad Bdair, Orabi Hajjeh, Mohammed AbuBaha, Waseem Shehadeh, Ameer Awashra, Ibrahim Alazizi, Raya Fuqha, Sakeena Saife, Hasan Fuqha, Fathi Milhem, Husam Hamshary, Dana Abuzahra, Umar Shuaib

**Affiliations:** ^1^ Department of Medicine, Faculty of Medicine and Health Sciences An‐Najah National University Nablus Palestine; ^2^ Center for Neurological Restoration, Neurological Institute, Cleveland Clinic Foundation Cleveland Ohio USA

**Keywords:** Aquaporin‐4, DTI‐ALPS, glymphatic system, neurodegeneration, perivascular spaces

## Abstract

**Background:**

The glymphatic system is a perivascular cerebrospinal fluid (CSF)–interstitial fluid (ISF) exchange pathway that supports brain homeostasis by clearing metabolic waste and neurotoxic proteins. Across central nervous system diseases, converging evidence indicates that glymphatic dysfunction represents a shared pathophysiological axis linking vascular, astroglial, inflammatory, and sleep‐related disturbances to impaired solute clearance.

**Results and Conclusion:**

In this review, we synthesize mechanistic and clinical evidence for glymphatic impairment in acute brain injury (ischemic and hemorrhagic stroke, traumatic brain injury) and chronic neurological disorders (Alzheimer's disease, Parkinson's disease, cerebral small vessel disease, multiple sclerosis, idiopathic normal pressure hydrocephalus, idiopathic intracranial hypertension, epilepsy, and headache disorders). Major mechanisms include (i) aquaporin‐4 (AQP4) depolarization/mislocalization at astrocytic endfeet, reducing perivascular water transport; (ii) perivascular space compression or obstruction from cytotoxic/vasogenic edema, blood‐derived products, protein aggregates, or altered extracellular matrix; (iii) loss of arterial pulsatility and vascular stiffening, weakening the driving forces for convective exchange; (iv) blood–brain barrier disruption and neuroinflammation, which remodel perivascular architecture and amplify clearance failure; and (v) sleep and autonomic dysregulation, including altered noradrenergic tone, which suppresses glymphatic activity during periods when clearance is normally maximal. Clinically, glymphatic dysfunction can be probed using diffusion tensor imaging–analysis along the perivascular space (DTI‐ALPS), contrast‐enhanced MRI approaches, and structural surrogates such as enlarged perivascular spaces, with emerging associations to cognition, mood, and disease severity. Finally, we discuss translational strategies aimed at restoring clearance, including sleep/circadian optimization, vascular risk control, anti‐inflammatory approaches, AQP4‐ and TRPV4‐oriented targets, and neuromodulation. Mechanism‐guided, standardized imaging and longitudinal interventional studies are needed to establish glymphatic biomarkers as actionable therapeutic and prognostic tools.

## Graphical abstract

Glymphatic Dysfunction in Neurological Disorders: The glymphatic system is a brain‐wide clearance system that removes neurotoxic waste via AQP4‐mediated CSF‐ISF exchange. Dysfunction through AQP4 mislocalization, BBB injury, and inflammation annihilates clearance and causes acute (stroke, TBI) and chronic disease. Lifestyle, sleep, and vascular interventions hold promise for restoring glymphatic function and improving neurological outcomes.

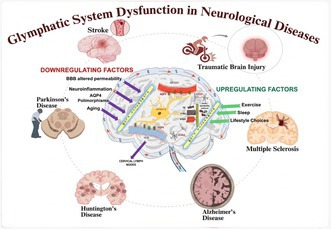

## 1 Introduction

**FIGURE 1:**
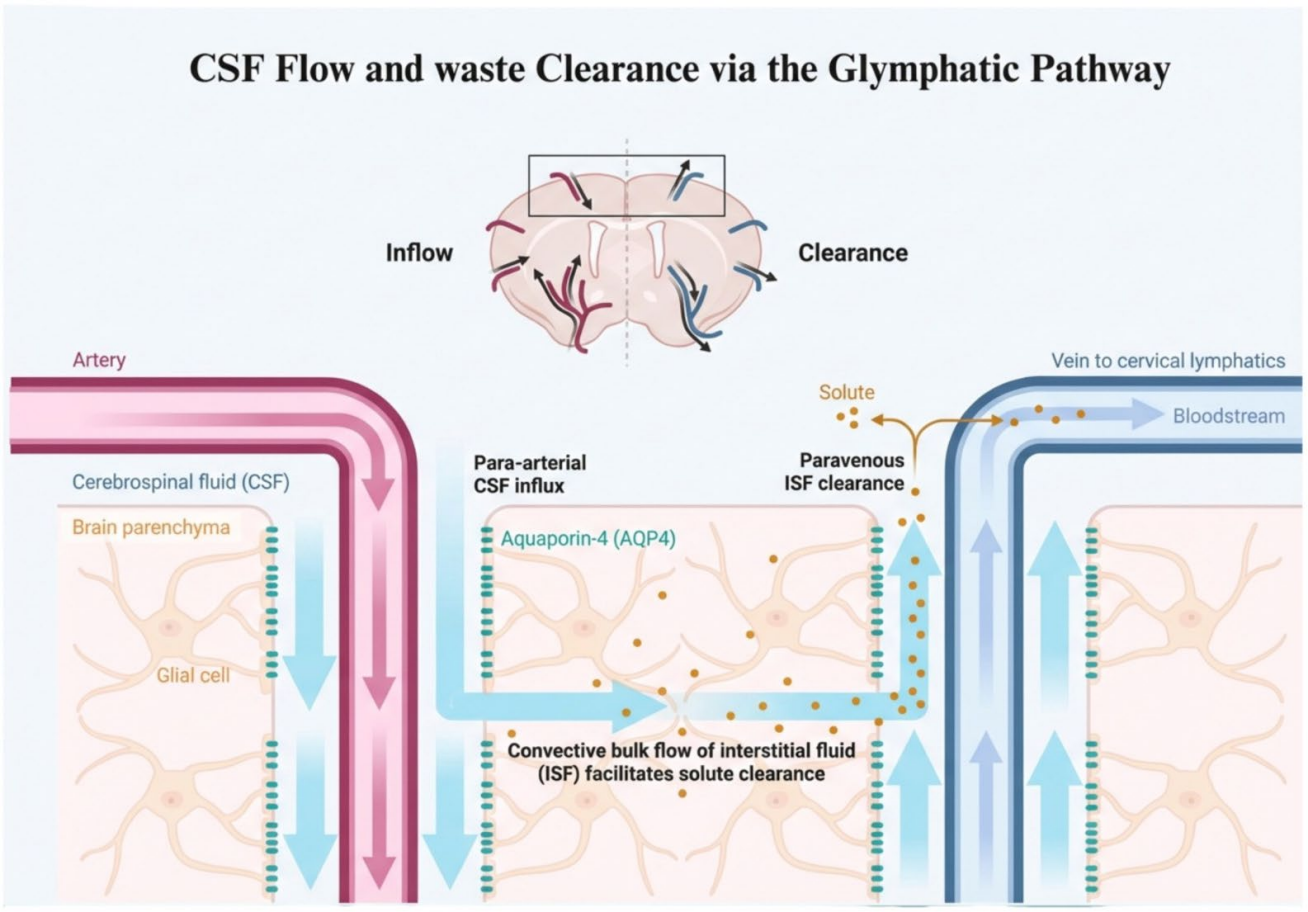
Glymphatic pathway of CSF flow and waste clearance. CSF enters the brain via para‐arterial routes, facilitated by aquaporin‐4 (AQP4) on astrocytes, mixes with interstitial fluid (ISF), and clears solutes through perivenous pathways toward the cervical lymphatics. Created in https://BioRender.com.

Historically, the central nervous system (CNS) was considered to be devoid of a system to manage its metabolic waste products, similar to the lymphatic system's role in managing waste products throughout the rest of the body [1, 2]. In 2012, the glymphatic system was first described as a glia‐dependent clearance pathway that redefined our understanding of neurofluid dynamics and brain homeostasis [3, 4, 5]. This system functions through a network of perivascular spaces (PVSs) that facilitates the passage of cerebrospinal fluid (CSF) from the subarachnoid space into the brain's interstitium, modulated by aquaporin‐4 (AQP4) channels in the astrocytes surrounding the PVSs. Mixing with the interstitial fluid (ISF) facilitates the clearance of solutes. Subsequently, the CSF‐ISF fluid and the waste products collected along the way get drained into the perivenous spaces, reaching the meningeal lymphatic system and, finally, the cervical lymph nodes [2, 3, 5, 6, 7]. (Figure 1).

Beyond its structural uniqueness, the glymphatic system has a pivotal role in physiologically maintaining brain health and clearing neurotoxic waste products from the interstitial space [3]. Some of the most important solutes eliminated through this pathway are amyloid‐β, α‐synuclein, and tau proteins, which are implicated in the pathogenesis of numerous neurodegenerative disorders such as Alzheimer's disease [3, 5, 8, 9, 10]. Experimental studies have shown that glymphatic transport is most active during sleep, when the interstitial space expands and CSF influx increases, facilitating more efficient clearance [4, 10]. On the other hand, the disruption of aquaporin‐4 (AQP4) polarization or sleep deprivation significantly decreases this clearance, resulting in the accumulation of pathological proteins [5, 10, 11]. Thus, the glymphatic system plays a pivotal role in linking sleep, cellular waste management, and long‐term cognitive health.

Recent research suggests that glymphatic dysfunction might have a role in the pathophysiology of acute and chronic neurological conditions [3, 8, 9]. For instance, in stroke, ischemia and hemorrhage have been shown to cause collapse of perivascular spaces and redistribution of AQP4 away from astrocytic endfeet, preventing CSF‐ISF exchange and promoting edema and secondary injury [12, 13, 14]. Similarly, in neurodegenerative diseases such as Alzheimer's disease and Parkinson's disease, impaired glymphatic flow is associated with the accumulation of misfolded proteins, likely because of age‐related vascular changes and chronic AQP4 mislocalization [5, 6, 9]. Collectively, these findings emphasize the transitional importance of the glymphatic system, with its potential therapeutic implications for restoring waste clearance mechanisms, optimizing sleep, and modulating astroglial physiology in disease prevention.

In this review article, we talk about the glymphatic system in depth, starting with explaining the normal glymphatic physiology and homeostasis and how it is affected in certain diseases such as stroke and how its dysfunction is complicit in the occurrence of other diseases such as Alzheimer's disease and Parkinson's disease, then discuss the diagnostic tools that can be used to assess glymphatic function, and finally suggest how it may be used in therapeutics and future directions in glymphatic system research.

## 2 Mechanisms of Glymphatic Dysfunction

Aquaporin‐4 (AQP4), a water channel primarily located at the endfeet of astrocytes, plays an integral part in facilitating fluid movement. In healthy physiology, AQP4 polarization ensures efficient glymphatic flow, enabling rapid clearance of neurotoxic solutes such as lactate, amyloid‐beta (Aβ), and tau proteins. The glymphatic system is a recently characterized brain‐wide perivascular network that oversees the removal of metabolic waste from the interstitial space through convective exchange between cerebrospinal fluid (CSF) and interstitial fluid (ISF). This clearance pathway is primarily active during sleep [5].

AQP4 plays a crucial role in glymphatic transfer. It has been shown in experimental models that AQP4 mislocalization or deletion impairs the flow of CSF into the brain parenchyma and significantly slows the elimination of interstitial solutes. AQP4 failure has been directly associated with decreased clearance of tau and Aβ in models of Alzheimer's disease (AD), which promotes pathological accumulation and ensuing cognitive impairments. Its importance in preserving the homeostatic brain environment is further highlighted by the fact that AQP4 depolarization, associated with aging or inflammation, exacerbates this clearance failure [15, 16], further emphasizing its importance in preserving brain homeostasis.

In the brain parenchyma, neurotoxic chemicals start to build up when the glymphatic system is defective. This accumulation of metabolic waste fuels inflammation, oxidative stress, and microglial activation, leading to neurodegeneration. Furthermore, protein aggregates, such as tau and Aβ, can further restrict perivascular spaces, exacerbating this dysfunction and generating a vicious cycle of decreased clearance and worsening injury [9].

Glymphatic function is significantly impaired in the setting of stroke, particularly acute ischemic stroke. This impairment is primarily driven by cytotoxic edema, which compresses perivascular pathways and thereby effectively halts glymphatic flow in affected regions. In addition, arterial occlusion reduces the pulsatile driving forces necessary for glymphatic transport. Dysregulated astrocytic AQP4 expression may initially limit edema formation; however, over time it contributes to abnormal water dynamics and impaired waste clearance. Importantly, as edema resolves and cerebral perfusion improves, glymphatic function may progressively recover, suggesting a dynamic and potentially reversible dysfunction rather than permanent impairment [17].

Astrocytes are involved in both blood–brain barrier (BBB) control and glymphatic function. During a stroke, AQP4 mislocalization occurs when reactive astrocytes lose their polarity following ischemic damage, which damages the BBB's structural integrity and impairs CSF–ISF exchange, allowing toxic plasma components to enter brain tissue. Neuronal damage is exacerbated by the ensuing inflammation and vasogenic edema. Thus, astroglial responses and vascular integrity are closely associated with glymphatic dysfunction in stroke [18].

Chronic glymphatic dysfunction has been associated with many chronic stroke risk factors, including diabetes, age, and hypertension. These disorders hinder the transport of CSF by causing vascular stiffness and decreased arterial pulse. Additionally, AQP4 mislocalization occurs in these disorders, and glymphatic exchange is impeded by endothelial dysfunction and chronic systemic inflammation. The long‐term consequences of these risk factors on brain waste clearance pathways are supported by the finding that decreased glymphatic inflow in diabetes models has been associated with cognitive decline and higher susceptibility to neurodegenerative alterations, suggesting long‐term consequences [19].

Glymphatic dysfunction is a key pathogenic factor in neurodegenerative diseases such as Parkinson's and Alzheimer's disease. The accumulation of extracellular amyloid plaques and the subsequent loss of AQP4 polarity in AD prevent perivascular flow and decrease solute removal. Similarly, in PD, problems associated with AQP4 and meningeal lymphatic dysfunction are connected to the inability to clear α‐synuclein aggregates. These deficits lead to neuroinflammation and neuronal degeneration, compounding the effects of α‐synuclein accumulation [20].

Microvascular disease, sleep dysfunction, and aging‐related astroglial alterations are additional pathways that contribute to glymphatic failure in neurodegeneration. Glymphatic efficiency is diminished as a result of AQP4 polarization loss and decreased arterial compliance that occur with aging. Lack of sleep reduces the time when glymphatic transport is most active during slow‐wave activity, further impairing waste clearance. Clearance deficiencies are also exacerbated by neuroinflammation and small vessel disease, which alter the perivascular architecture. Individual susceptibility to glymphatic dysfunction is influenced by genetic variables, including polymorphisms in AQP4, which have been associated with an increased risk of dementia [20].

## 3 Clinical Relevance and Implications

There is growing evidence that problems with the glymphatic system play a role in the development and progression of several chronic neurodegenerative diseases [21]. In the sections below, we explore how this dysfunction may contribute to each disease.

### 3.1 Glymphatic Dysfunction in Chronic Neurodegeneration

#### 3.1.1 Alzheimer's Disease

AD is characterized by the accumulation of β‐amyloid and tau proteins. The glymphatic system is one mechanism by which the brain clears these proteins, and this may be impaired in patients with AD [16, 22]. Patients with AD have been found to have mislocalized or non‐polarized AQP4 channels, contributing to the accumulation of the neurotoxic proteins [23]. Arighi et al. measured AQP4 levels in cerebrospinal fluid (CSF) in 11 AD patients, 10 normal pressure hydrocephalus (NPH) patients, and nine controls, finding that AQP4 was significantly decreased in AD patients and tended to decrease in NPH patients [23, 24].

Recent human studies provided evidence of glymphatic dysfunction in AD through advanced neuroimaging modalities. The diffusion tensor imaging analysis along the perivascular space (DTI‐ALPS) index, a non‐invasive MRI biomarker of glymphatic flow, has shown decreased values in AD patients, indicating impaired perivascular efflux [25, 26].

Sleep disturbances are common among patients with AD, with approximately one‐third experiencing clinically documented sleep disorders, and represent an important contributor to glymphatic dysfunction. Glymphatic clearance mainly occurs during slow‐wave sleep. Therefore, most AD patients with sleep disturbances—particularly those with diminished slow‐wave sleep—are likely to experience further impaired glymphatic function [27]. In vivo studies have shown that the clearance activity of the glymphatic system is significantly increased in sleep compared to wakefulness, with its peak clearance occurring during slow‐wave sleep [28].

Patients with AD, especially those with comorbid sleep disorders, have higher CSF levels of orexin. Orexin antagonists have shown promise by increasing slow‐wave sleep and enhancing the glymphatic system clearance [29, 30]. In the same context, the use of focused acoustic stimulation to improve slow‐wave sleep highlights another therapeutic potential for enhancing protein clearance in AD [31, 32]. Additionally, AQP4 modulation is a key therapeutic target; although no AQP4 agonists are currently clinically available, preclinical efforts to correct AQP4 polarization and localization may help restore glymphatic function in AD patients [8].

In another noteworthy potential clinical application, a new minimally invasive surgery was done in China as a potential treatment for AD. Their hypothesis is creating an anastomosis between the lymphatic and venous systems in an attempt to enhance amyloid protein clearance [33]. While the initial report primarily describes the theoretical rationale and surgical feasibility, related exploratory clinical data from a prospective single‐arm cohort of patients undergoing deep cervical LVA demonstrated short‐term improvements in MMSE scores, with trends toward improvement in other cognitive measures and biomarkers, although without definitive efficacy or long‐term outcome data. These findings support the feasibility of LVA as a therapeutic concept but underscore the need for larger, controlled trials to establish its clinical effectiveness [34].

A recent study used the (DTI‐ALPS) index as an indicator of glymphatic function. It was found that higher values were associated with a delay in the conversion of mild cognitive impairment (MCI) to AD [35]. However, the correlation between changes in glymphatic flow over time and the progression from MCI to AD remains unclear.

#### 3.1.2 Parkinson's Disease

PD is characterized by aggregation of α‐synuclein proteins [36]. These form dense neuronal inclusions known as Lewy bodies, which correlate with disease progression. Recent evidence suggests that glymphatic dysfunction may contribute to PD pathogenesis via decreased α‐synuclein protein clearance and presumed reduced diffusion along nigro‐striatal perivascular channels [36, 37]. Additionally, REM sleep behavior disorders, which typically occur years before the onset of PD motor manifestations, are suggested to be markers of glymphatic dysfunction contributing to the pathogenesis, based on the sleep theory mentioned above [27, 28, 38, 39].

Autonomic dysfunction in PD may further hinder clearance of glymphatic flow via its effects on cerebrospinal fluid (CSF) pulsatility [40, 41, 42].

**FIGURE 2:**
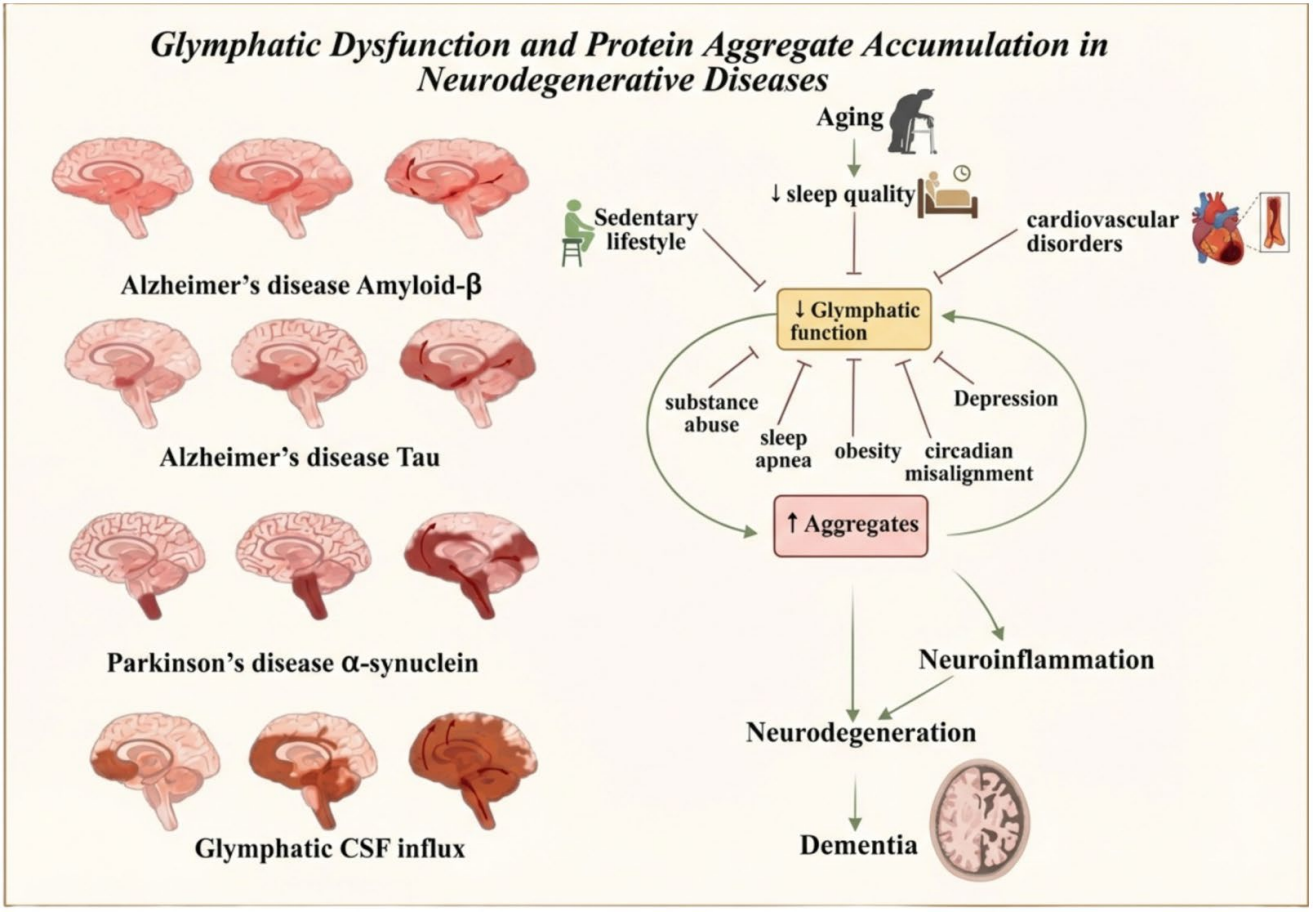
Glymphatic Dysfunction in Neurodegeneration, Impaired glymphatic clearance leads to accumulation of Amyloid‐β, Tau, and α‐synuclein in Alzheimer's disease and Parkinson's disease. Factors like aging, sleep disorders, and obesity worsen this dysfunction, promoting protein aggregation, neuroinflammation, and dementia. Created in https://BioRender.com.

Interestingly, some studies suggest that L‐DOPA, despite not being a disease‐modifying therapy, may help restore CSF dynamics, possibly by affecting blood vessel function; however, these findings are exploratory and not conclusive [43]. Overall, these observations highlight potential, co‐occurring pathophysiological processes that could inform future models of PD management and hypothesis‐driven studies focused on glymphatic‐related measures, rather than establishing a proven therapeutic pathway at this time. (Figure 2).

#### 3.1.3 Huntington's Disease and Amyotrophic Lateral Sclerosis

Although the literature is relatively scarce regarding the relationship between the glymphatic system in HD and ALS, recent studies highlight a possible correlation.

In HD, mutant huntingtin (mHTT) protein aggregates in proximity to astrocytes, overwhelming their capacity to maintain a functional glymphatic system [44]. Meanwhile, in a subtype of ALS, the accumulation of TDP‐43 may impair glymphatic function, either by directly injuring astrocytes or blocking the perivascular spaces [45].

Despite significantly diverse symptomatology, both diseases share a common pathophysiological theme, characterized by astroglial dysfunction and proteinopathy, which leads to impaired clearance of neurotoxic substrates [44, 45]. This shared theme underscores the potential of glymphatic imaging biomarkers and therapeutic modulation as novel tools in these devastating neurodegenerative diseases. However, despite these mechanistic insights, evidence supporting the use of glymphatic dysfunction as a reliable prognostic biomarker in HD and ALS remains limited. In ALS, recent longitudinal studies using diffusion‐based glymphatic measures (e.g., DTI–ALPS) suggest an association with clinical severity and disease progression, but findings remain heterogeneous and require further validation in larger cohorts. In HD, available human data are largely cross‐sectional, highlighting glymphatic impairment rather than establishing prognostic utility [46, 47].

### 3.2 The Glymphatic System and Traumatic Brain Injury

A new and emerging area of study in which the glymphatic system is being investigated is traumatic brain injuries (TBI). Various secondary processes and damage begin shortly after the primary traumatic injury to the brain, ranging from inflammation and hypoxia to altered local metabolism. These secondary processes alter the normal function of astrocytes as well as the localization and expression of AQP4 channels. This causes astrocyte swelling and the delocalization of AQP4 following TBI, leading to the breakdown of normal structure and glymphatic impairment, as well as the formation of cytotoxic edema [48, 49].

TBI also causes obstruction of perivascular spaces and consequent reduction of overall glymphatic flow and clearance via different mechanisms. After sustaining a TBI, there is a surge of norepinephrine that acts on the glymphatic system, resulting in a decrease in flow and clearance of proteins, solutes, and cellular debris, which subsequently accumulate in perivascular spaces [50, 51]. Studies aimed at improving impaired glymphatic flow found that doing so was associated with reduced secondary injury, including decreased edema and neuroinflammation, more efficient clearance of proteins and cellular debris, and subsequently better neurological and cognitive recovery, supporting its role as both a marker and a driver of TBI‐associated dysfunction [51]. Patients suffering from TBI also commonly have sleep disturbances, which independently cause glymphatic dysregulation [52].

Chronic or repeated TBI was also found to affect the brain's cognitive and executive functions, as well as its structure. Several studies have pointed to how TBI and its subsequent effects on the glymphatic system lead to cognitive dysfunction that is commonly progressive in nature, and this is now thought to be one of the prominent theories as to why TBI leads to delayed cognitive dysfunction in diseases such as chronic traumatic encephalopathy and other forms of dementia [53, 54, 55]. Jung et al., 2024 reported that former American football players had larger MRI‐visible white matter perivascular space (WM‐PVS) volume than unexposed controls, with WM‐PVS volume expressed as the fraction of the whole white matter, and that higher WM‐PVS volume correlated with greater repetitive head‐impact exposure and worse cognitive performance, indicating a generalized white matter effect rather than a focal regional predilection [55]. Having a history of remote brain trauma was also linked to worse cognitive and executive functions [53].

New and emerging imaging techniques and protocols are being developed to study glymphatic dysfunction following traumatic brain injury (TBI). For instance, new techniques using T2‐weighted MRI images are being studied to quantify enlarged perivascular spaces in TBI patients [56, 57]. Other techniques include using diffusion tensor imaging (DTI) and applying it in a certain way to calculate what is called the Analysis aLong the Perivascular Space (ALPS) index, giving birth to what is now known as DTI‐ALPS. This method measures glymphatic function and flow, serving as an indicator in various neurologic diseases, including TBI [58, 59, 60, 61].

Following recent discoveries implicating the glymphatic system in post‐traumatic brain injury (TBI) complications, new therapies are being developed to manage the glymphatic aspect of these complications. After examining the fact that the glymphatic system is primarily active during sleep, a new form of non‐invasive therapy for TBI has been proposed, termed circadian therapy. This therapy aims to optimize sleep through pharmacologic and behavioral or light based measures, including melatonin 3 to 5 mg at bedtime, morning blue light therapy for about 30 min within 2 h of awakening, evening blue light restriction with screen time limits and blue blocking glasses, screening and treatment of sleep apnea with continuous positive airway pressure, and adjunctive omega 3 fatty acids, each reported to improve sleep architecture and glymphatic function in concussion and traumatic brain injury cohorts [52]. Other studies examine the use of interleukin‐33 or glucagon‐like peptide 1 (GLP‐1) receptor agonists after noticing their effects on AQP4 channels, which were found to reduce edema, enhance waste clearance and improve overall outcomes [62]. Other studies have also examined the potential use of very low‐intensity ultrasound as a method of enhancing glymphatic circulation and waste clearance by modulating the TRPV4‐AQP4 pathway [63]. Another route explored by some studies is the use of adrenergic blockers, such as prazosin, propranolol, and atipamezole, in order to offset the norepinephrine‐induced glymphatic blockage after TBI [51].

### 3.3 The Glymphatic System and Stroke

**FIGURE 3:**
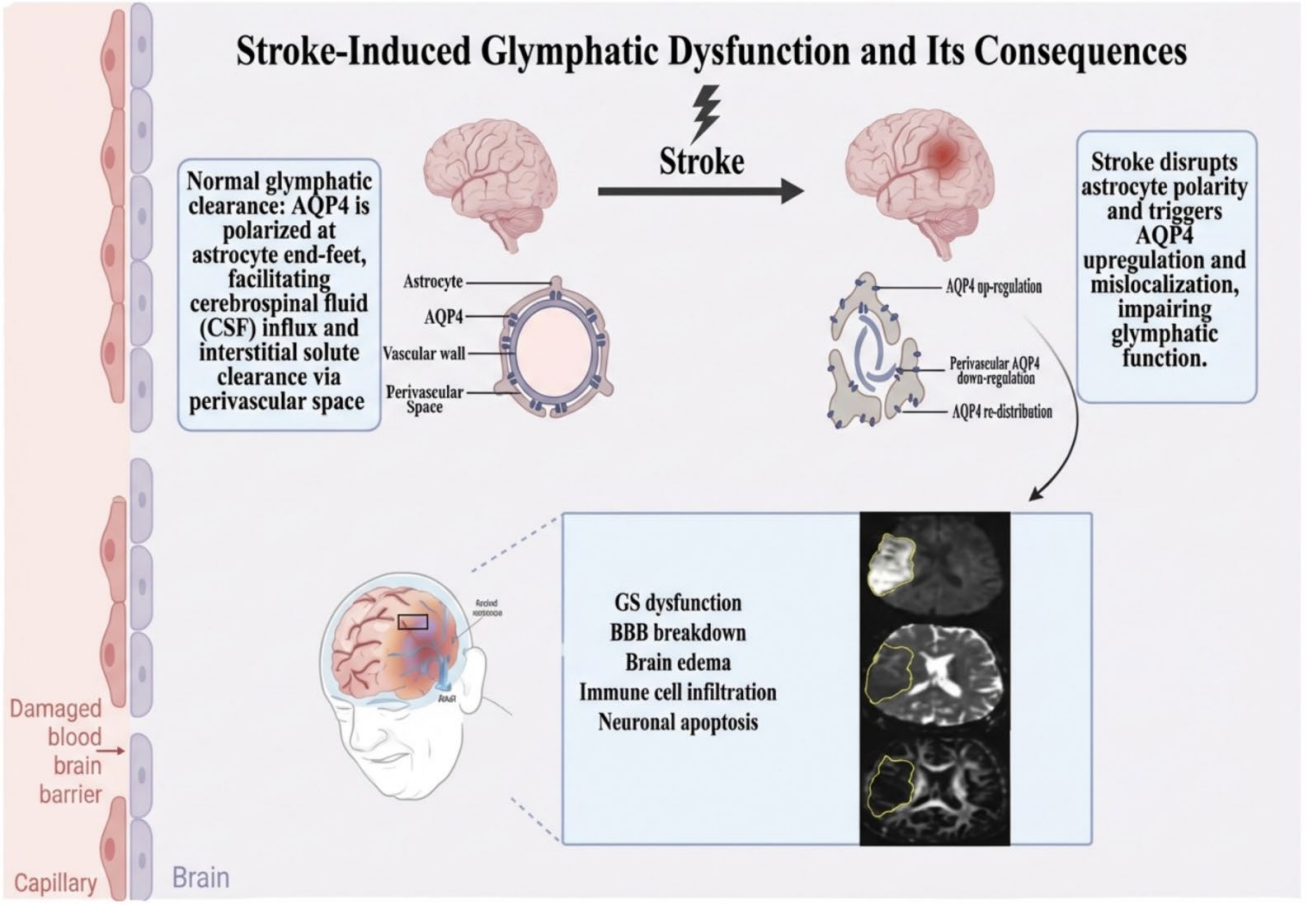
Stroke‐Induced Glymphatic Impairment. Stroke disrupts AQP4 polarity in astrocytes, reducing glymphatic clearance. This leads to edema, blood–brain barrier breakdown, immune cell infiltration, and neuronal damage. Created in https://BioRender.com.

Stroke causes glymphatic dysfunction via several mechanisms. First, in hemorrhagic stroke, the glymphatic flow is impaired primarily due to blood components, chiefly fibrin and fibrinogen deposits [19]. In ischemic stroke, it is thought that after injury the glymphatic system facilitates increased inflow of cerebrospinal fluid while causing a decreased outflow of interstitial fluid, leading to cerebral edema, which is driven by TRPV4‐induced depolarization of AQP4 [12, 64]. Research shows that post‐stroke glymphatic dysregulation is related to cognitive dysfunction and depressive symptoms [65, 66]. Tracking this dysregulation via imaging has been potential to be a possible predictor of cognitive impairment [66, 67]. There have also been studies exploring the possibilities of using enlarged perivascular spaces as potential indicators and risk stratifiers of epilepsy incidence in stroke patients; however, results remain inconclusive [14]. The aforementioned findings have also caused researchers to consider therapies targeting components of the glymphatic pathway as future options for post‐stroke management [68]. (Figure 3; Table 1).

**TABLE 1:** Glymphatic dysfunction in acute and vascular neurological diseases.

Diseases	Pathophysiologic impact	Representative imaging marker	Clinical and therapeutic implications
Stroke	AQP4 depolarization and TRPV4 activation impair CSF–ISF exchange, causing edema; fibrin deposits and vascular stiffness restrict perivascular flow	MRI shows reduced glymphatic clearance correlating with cognitive and mood decline	Maintain AQP4 polarity (MMP‐9 or TRPV4 inhibition); improve perfusion and sleep; consider neuromodulation (VNS, tDCS)
Traumatic brain injury(TBI)	Astrocytic AQP4 mislocalization and norepinephrine surge suppress glymphatic inflow; perivascular obstruction leads to protein debris and inflammation	Enlarged perivascular spaces and reduced ALPS index; recovery parallels cognitive improvement	Optimize sleep and circadian rhythm; use adrenergic blockers, GLP‐1 agonists, or low‐intensity ultrasound
Idiopathic normal pressure hydrocephalus (iNPH)	Perivascular drainage failure causes ventricular dilation, in addition to cognitive and gait impairment	Delayed gadolinium clearance and lower ALPS index; partial normalization after shunt surgery	Shunting restores glymphatic flow; ALPS index can monitor postoperative cognitive response
Idiopathic intracranial hypertension (IIH)	Venous sinus stenosis and altered AQP4 function delay glymphatic clearance, retaining waste in limbic and olfactory regions	Delayed contrast washout and decreased ALPS values proportional to papilledema grade	Recognize glymphatic impairment as a contributor; use ALPS for diagnosis and follow‐up
Migraine/Headache disorders	Sleep‐related clearance reduction and CGRP‐driven vascular reactivity disturb perivascular exchange	Mixed ALPS results; enlarged perivascular spaces in centrum semiovale and midbrain	Enhance sleep hygiene and circadian alignment; explore CGRP modulation and standardize glymphatic imaging

Abbreviations: AQP4, Aquaporin‐4; CGRP, Calcitonin Gene‐Related Peptide; DTI‐ALPS, Diffusion Tensor Image Analysis along the Perivascular Space; GLP‐1, Glucagon‐Like Peptide‐1; PVS, Perivascular Spaces; tDCS, Transcranial Direct Current Stimulation; VNS, Vagus Nerve Stimulation.

### 3.4 The Glymphatic System and Small Vessel Disease

The glymphatic system also plays a role in cerebral small vessel disease. Research examining the relation of the glymphatic system to the pathogenesis of small vessel disease of the brain uncovered several possible pathways. In cerebral amyloid angiopathy, it is believed that decreased glymphatic flow and impaired protein clearance lead to the deposition of material in surrounding structures, such as vessel walls, thereby contributing to the disease [69]. This is not limited to amyloid angiopathy but also applies to other forms of small vessel disease, such as lacunar infarcts and microbleeds [70]. Studies demonstrated that the severity and recurrence of cerebral amyloid angiopathy, as well as related cognitive and executive dysfunction in patients, displayed a significant relation to ALPS index‐tracked glymphatic dysfunction [70, 71]. In other studies, it was shown that the pathogenesis of cerebral small vessel disease and glymphatic dysfunction are intertwined and that they share many risk factors, such as hypertension, diabetes, and aging, among many others. In the case of diabetes and hypertension, it has been shown that they contribute to glymphatic dysfunction by interfering with the normal pulsatility of cerebral arteries, which is the main driving force of the glymphatic system [72].

### 3.5 The Glymphatic System and Other Neurological Diseases

#### 3.5.1 Glymphatic System and Multiple Sclerosis

Recent studies suggest the glymphatic system may be linked to the development and progression of multiple sclerosis (MS).

A recent cross‐sectional study demonstrates a potential link between MS and impaired glymphatic function by measuring ALPS‐index in patients with relapsing–remitting MS. Their findings demonstrate that patients have a significantly lower ALPS index compared to healthy controls, indicating reduced glymphatic function. Moreover, ALPS index has a negative correlation with expanded disability status scale (EDSS) scores, lesion burden, and brain atrophy, suggesting that impaired glymphatic flow may be associated with increased neurological disability and demonstrate that this may not only be a consequence of chronic neuroinflammation in MS but could also contribute to disease progression by hindering clearance of immune cells, inflammatory mediators, and metabolic waste [73]. These findings support the growing hypothesis that glymphatic dysfunction may play an active role in the pathogenesis and progression of MS, rather than being a secondary consequence of neuroinflammation alone.

Another cross‐sectional study demonstrates a significant inverse relationship between choroid plexus (CP) volume and the DTI‐ALPS index, suggesting a potential mechanistic link between CP pathology and impaired waste clearance in the central nervous system. This demonstrates that DTI‐ALPS partially explains the association between CP enlargement and both lesion burden and regional brain volume loss, supporting the hypothesis that glymphatic dysfunction may serve as a conduit through which CP‐related inflammation contributes to neurodegeneration in MS [74]. Moreover, the partial mediation also suggests that additional, glymphatic‐independent mechanisms likely contribute to MS‐related brain damage. These findings underscore the importance of the CP‐glymphatic axis in MS pathophysiology, suggesting that targeting these pathways may hold therapeutic potential [74].

#### 3.5.2 Evidence of Glymphatic Dysfunction in Idiopathic Normal Pressure Hydrocephalus (iNPH)

iNPH is a neurological disorder characterized by gait disturbance, dementia, and urinary incontinence. Recent research suggests that impaired glymphatic clearance may contribute to its pathophysiology. Recent studies have shown glymphatic impairment in iNPH. Ringstad et al. used intrathecal gadobutrol and MRI to track CSF flow in iNPH patients, finding delayed tracer influx and clearance, indicating disrupted glymphatic function [75]. Follow‐up studies confirmed these impairments were stable over time. Although age differences between patients and controls complicate interpretation, these findings suggest disease‐specific glymphatic deficits rather than aging alone [75]. Another study in 2022 demonstrated reduced glymphatic clearance in the entorhinal cortex (ERC), a region affected early in dementia, linking glymphatic dysfunction to memory decline. This suggests ERC dysfunction may be a hallmark of dementia progression in both iNPH and AD [76, 77].

In a study in 2019 that used DTI‐ALPS, ALPS index scores were significantly lower in iNPH than in healthy controls and than in a radiographic NPH‐mimicking condition characterized by ventriculomegaly due to cerebral atrophy (hydrocephalus ex vacuo) [78]. The lower ALPS indices observed in iNPH relative to ex vacuo ventriculomegaly suggest that impaired glymphatic function is a fundamental and measurable component specific to iNPH pathology, which may contribute to diagnostic criteria and constitute a potential therapeutic target. Most importantly, the inverse correlation between the ALPS index and ventricular volume appears to be specific to true idiopathic normal pressure hydrocephalus rather than a general characteristic of ventriculomegaly [78].

An observational longitudinal study in 2024 utilized (DTI‐ALPS) to measure glymphatic function in iNPH patients before and after shunt surgery [79]. The study included 35 patients with iNPH and 40 healthy controls who underwent MRI and neurocognitive assessments, but just 15 patients were reassessed for 3 months post‐shunt surgery. Results revealed that ALPS indexes were prominently decreased in iNPH patients compared to healthy individuals, and this index was negatively correlated with ventricular volume, suggesting impaired glymphatic clearance in the disease state [79].

Notably, the study demonstrated a significant increase in the ALPS index postoperatively, which indicates partial restoration of glymphatic function after CSF diversion. This improvement was associated with cognitive gains, as changes in the ALPS index were positively correlated with increases in Mini‐Mental State Examination (MMSE) scores. Moreover, the partial normalization of ALPS indices postoperatively but still lower than in healthy controls suggests that long‐term alterations in perivascular pathways may persist despite shunt placement [80]. Emerging evidence further suggests that diffusion‐based glymphatic measures, including the ALPS index, may carry predictive value for shunt responsiveness, with lower preoperative ALPS values and greater postoperative increases observed predominantly in clinically responsive patients [81, 82]. Collectively, these observations support glymphatic dysfunction as a core component of iNPH pathology, contributing to symptom development and progression, while highlighting its potential relevance as a biomarker of treatment response.

#### 3.5.3 Glymphatic System and Idiopathic Intracranial Hypertension (IIH)

IIH is a condition marked by elevated intracranial pressure without a clear cause. Emerging evidence suggests that dysfunction in the glymphatic system may play a role in altered cerebrospinal fluid dynamics in IIH. This was demonstrated in a cross‐sectional study investigating glymphatic function in 15 patients with IIH compared to 15 age‐ and sex‐matched controls. Using intrathecal administration of a contrast agent, the researchers observed that IIH patients exhibited increased retention and delayed clearance of the contrast compared to healthy controls. This abnormal retention was particularly evident in regions such as the frontal and temporal lobes, hippocampus, and olfactory bulb areas, which are closely associated with memory, cognition, and olfactory processing [80]. Overall, this research suggests that glymphatic dysfunction could be a key factor linking intracranial hypertension with neurological deficits [80].

Another retrospective study evaluated glymphatic clearance in 99 patients clinically diagnosed with idiopathic intracranial hypertension (IIH) compared to 6 normal healthy individuals using DTI‐ALPS [83]. The study found that patients with IIH had significantly impaired glymphatic clearance compared to healthy controls. The degree of glymphatic dysfunction correlated with clinical severity, which was assessed by Frisen papilledema grade and lumbar puncture opening pressure. Patients with higher papilledema grades and elevated opening pressures exhibited the lowest glymphatic clearance [83]. These findings reinforce the hypothesis that impaired glymphatic function plays a key role in IIH pathophysiology, potentially driven by venous sinus stenosis and altered aquaporin‐4 function. Despite limitations such as the small control group and retrospective design, the study suggests that DTI‐ALPS is a promising tool for diagnosis and disease monitoring in IIH. Future prospective studies with larger cohorts are warranted to validate these findings and explore underlying molecular mechanisms [83].

#### 3.5.4 Glymphatic System and Headache (Migraine and Others)

Migraine is a common neurological disorder often associated with altered brain homeostasis. One pilot study using the DTI‐ALPS index found no significant differences in glymphatic activity between healthy individuals and those with migraine, regardless of aura status, suggesting limited involvement of the glymphatic system in migraine pathogenesis [84]. In contrast, MRI studies have shown that enlarged perivascular spaces, particularly in the centrum semiovale and midbrain, are associated with migraine, serving as independent predictive markers, although not directly linked to clinical manifestations or chronification [85].

Zhang et al. reported an unexpected increase in the DTI‐ALPS index in patients with chronic migraine (CM), potentially reflecting altered vascular reactivity due to prolonged CGRP release. This increase was lateralized to the right hemisphere and may be tied to central sensitization mechanisms. However, the study's small sample size, limited glymphatic system assessment regions, and lack of CGRP quantification present limitations [86]. In contrast, Wu et al. demonstrated a negative correlation between the DTI‐ALPS index and migraine severity in CM, especially in cases with medication overuse, indicating impaired glymphatic and meningeal lymphatic function. Additionally, DTI‐ALPS values were inversely related to migraine disability and attack frequency, with DCE‐MRI findings further supporting lymphatic dysfunction in CM [87].

Although chronic migraine (CM) is often discussed in the context of impaired brain clearance, one study reported a higher (right‐dominant) DTI‐ALPS index in CM compared with episodic migraine and controls, suggesting an *apparent* increase in perivascular water diffusivity during migraine chronification [86]. Importantly, DTI‐ALPS is an indirect diffusion‐based surrogate, and its values can be influenced by factors that are not specific to convective “glymphatic clearance,” including vascular reactivity/vasomotion, perivascular geometry (e.g., enlarged perivascular spaces), and white‐matter microstructural changes; therefore, a higher ALPS index should not be interpreted as unequivocally “better glymphatic clearance” [88]. One plausible explanation is that migraine chronification alters neurovascular dynamics (e.g., sustained trigeminovascular activation and CGRP‐linked vasodilation/vasomotion), which may increase perivascular water mobility and thereby elevate ALPS as a concomitant neurovascular phenomenon rather than a compensatory restoration of waste clearance [89]. In contrast, a separate cohort integrating DTI‐ALPS with dynamic imaging of lymphatic/meningeal outflow reported lower ALPS values and impaired glymphatic/meningeal lymphatic function in CM, particularly in medication‐overuse headache, with associations to attack burden/disability [87]. Collectively, these findings suggest that migraine‐related “glymphatic signatures” may be phenotype‐ and state‐dependent (e.g., medication overuse, sleep disruption, autonomic/vascular tone) and may reflect neurovascular–meningeal lymphatic coupling rather than a single linear trajectory of “dysfunction.” Notably, emerging clinical evidence indicates that baseline migraine phenotypes and neurovascular features, closely linked to CGRP‐mediated trigeminovascular activity, may be associated with therapeutic response to CGRP‐pathway inhibitors, raising the possibility that perivascular or glymphatic‐related markers could have predictive value for treatment stratification, pending confirmation in prospective multimodal imaging studies [90].

Other investigations have examined the role of glymphatic system dysfunction in white matter hyperintensities (WMHs), which are common in migraine. While some hypothesize that impaired CSF‐ISF outflow and spreading ischemia from cortical spreading depression contribute to WMHs [70, 91, 92, 93, 94, 95]. Ornello et al. did not find an association between glymphatic system dysfunction and WMHs in migraines using the DTI‐ALPS index [96]. Overall, findings are inconsistent, with methodological limitations and heterogeneous study populations hindering generalizability. Standardized protocols and multicenter designs are necessary to clarify the involvement of the glymphatic system in migraine.

Beyond migraine, glymphatic system dysfunction has been implicated in other headache disorders. The bidirectional link between sleep disturbances and glymphatic system impairment is particularly relevant, as disrupted glymphatic flow during poor sleep may lead to the accumulation of pro‐inflammatory and neuroexcitatory molecules that contribute to headache generation [39, 97, 98, 99, 100]. Cluster headache (CH), characterized by severe unilateral pain and autonomic symptoms, has been associated with reduced glymphatic system activity, especially in older individuals [19, 101, 102, 103, 104, 105].

#### 3.5.5 Glymphatic System and Seizure

Seizures are associated with abnormal neuronal activity and disrupted brain homeostasis. Recent research suggests that seizures may impair glymphatic clearance, leading to the accumulation of neurotoxic waste.

A recent retrospective study investigated the role of glymphatic system dysfunction in focal epilepsy using the DTI‐ALPS method. The study included 100 patients with focal epilepsy who had normal structural MRI findings and 79 age‐ and sex‐matched healthy controls. Among the patients, 38 were classified as poor responders and 62 as good responders to anti‐seizure medications (ASMs), based on seizure control at the time of imaging [106]. The glymphatic function, quantified using the DTI‐ALPS index, was significantly reduced in the epilepsy group compared to healthy controls. Furthermore, poor ASM responders showed a considerably lower DTI‐ALPS index than good responders. The study also found a significant negative correlation between the DTI‐ALPS index and both patient age and duration of epilepsy, suggesting a potential association between impaired glymphatic clearance and both the development of epilepsy and resistance to pharmacologic treatment [106].

Another cross‐sectional study investigated the role of glymphatic system dysfunction in semantic verbal fluency (SVF) impairment among patients with unilateral temporal lobe epilepsy (TLE). Using DTI‐ALPS, the researchers found that the ipsilateral hemisphere showed significantly reduced glymphatic clearance compared to the contralateral side. Both decreased DTI‐ALPS index and increased choroid plexus volume (CPV/ICV) emerged as independent risk factors for SVF impairment. Mediation analyses further revealed that the DTI‐ALPS index fully mediated the relationship between CP enlargement and SVF performance, suggesting that impaired glymphatic drainage may be a key mechanism underlying cognitive dysfunction in TLE. These findings highlight the potential of targeting glymphatic clearance rather than CSF production as a therapeutic approach to mitigate semantic memory deficits in epilepsy [107]. (Table 2).

**TABLE 2:** Glymphatic dysfunction in neurodegenerative and inflammatory diseases.

Diseases	Pathophysiologic impact	Representative imaging marker	Clinical/Therapeutic implications
Alzheimer's Disease (AD)	AQP4 mislocalization impairs β‐amyloid and tau clearance; reduced slow‐wave sleep suppresses glymphatic inflow and promotes protein buildup	Lower DTI‐ALPS index and CSF AQP4 levels; ALPS decline correlates with gray matter loss and cognition	Improve sleep (orexin antagonists, acoustic stimulation); target AQP4; consider lymphatic–venous bypass
Parkinson's Disease (PD)	α‐Synuclein aggregation and autonomic dysfunction reduce CSF pulsatility and perivascular drainage	Lower ALPS index; meningeal lymphatic dysfunction evident on MRI	Optimize sleep and autonomic tone; assess L‐DOPA effects on CSF flow; integrate ALPS in progression studies
Multiple Sclerosis (MS)	Impaired immune and metabolite clearance; choroid plexus enlargement disrupts CSF–ISF exchange	Reduced ALPS correlates with EDSS, lesion burden, and atrophy; mediates CP–atrophy relationship	Target the CP–glymphatic axis; use ALPS as a biomarker of disability and inflammation
Epilepsy (TLE/Focal)	Perivascular obstruction and CP enlargement reduce drainage and provoke inflammation; sleep loss aggravates clearance failure	Lower ALPS index in poor responders; mediates CP–verbal fluency link	Promote sleep and drainage; use ALPS to monitor cognition and treatment response
Huntington's/ALS	Protein aggregates injure astrocytes and narrow perivascular spaces, halting glymphatic flow	Animal and early human data show reduced glymphatic influx and clearance	Investigate AQP4‐modulating and flow‐restoring agents; develop longitudinal ALPS studies for early detection

Abbreviations: AQP4, Aquaporin‐4; ASM, Anti‐Seizure Medication; CGRP, Calcitonin Gene‐Related Peptide; CP, Choroid Plexus; CSF, Cerebrospinal Fluid; DTI‐ALPS, Diffusion Tensor Image Analysis along the Perivascular Space; EDSS, Expanded Disability Status Scale; GLP‐1, Glucagon‐Like Peptide‐1; ISF, Interstitial Fluid; PVS, Perivascular Spaces; tDCS, transcranial Direct Current Stimulation; TLE, Temporal Lobe Epilepsy; VNS, Vagus Nerve Stimulation.

## 4 Diagnostic Tools for Assessing Glymphatic Function

Building upon the clinical relevance and implications of glymphatic dysfunction in stroke and neurodegenerative diseases, accurate evaluation is necessary to enhance our understanding of the glymphatic function's contribution to these conditions. Multiple diagnostic techniques based on imaging have been created, each providing a different perspective on this complex clearing system. Intrathecal contrast‐enhanced MRI is one of the simplest methods; it uses lumbar puncture to infuse gadolinium‐based contrast agents to track the flow of cerebrospinal fluid (CSF) along glymphatic routes. High‐resolution, time‐dependent imaging of CSF‐interstitial fluid exchange is possible with this approach; however, its therapeutic use is limited by its invasiveness and probable toxicity [108].

Intravenous contrast MRI, which involves the injection of gadolinium into a peripheral vein, has been suggested as a less invasive option but lacks accuracy. A potential, albeit still less accurate, diagnostic tool is delayed imaging sequences, which can provide indirect indicators of glymphatic transport, even though they do not allow direct monitoring of perivascular flow [109].

More recently, the ALPS index has made DTI a non‐invasive technique for evaluating glymphatic function. This method provides a reproducible imaging biomarker that is correlated with glymphatic efficiency by quantifying the directionality of water diffusion along white matter tracts and perivascular spaces [110]. It has been demonstrated that the DTI‐ALPS index is helpful in detecting changes in glymphatic dynamics in diseases like Alzheimer's and aging [103]. Structural indicators, including enlarged perivascular spaces (ePVS), have drawn interest in addition to functional imaging. Despite being non‐specific, they can be visualized on standard T2‐weighted MRI scans and are increasingly considered an indirect indicator of poor glymphatic clearance, especially when they are prominently situated in the centrum semiovale or basal ganglia [111]. There are numerous studies that link ePVS with neurodegenerative diseases.

**FIGURE 4:**
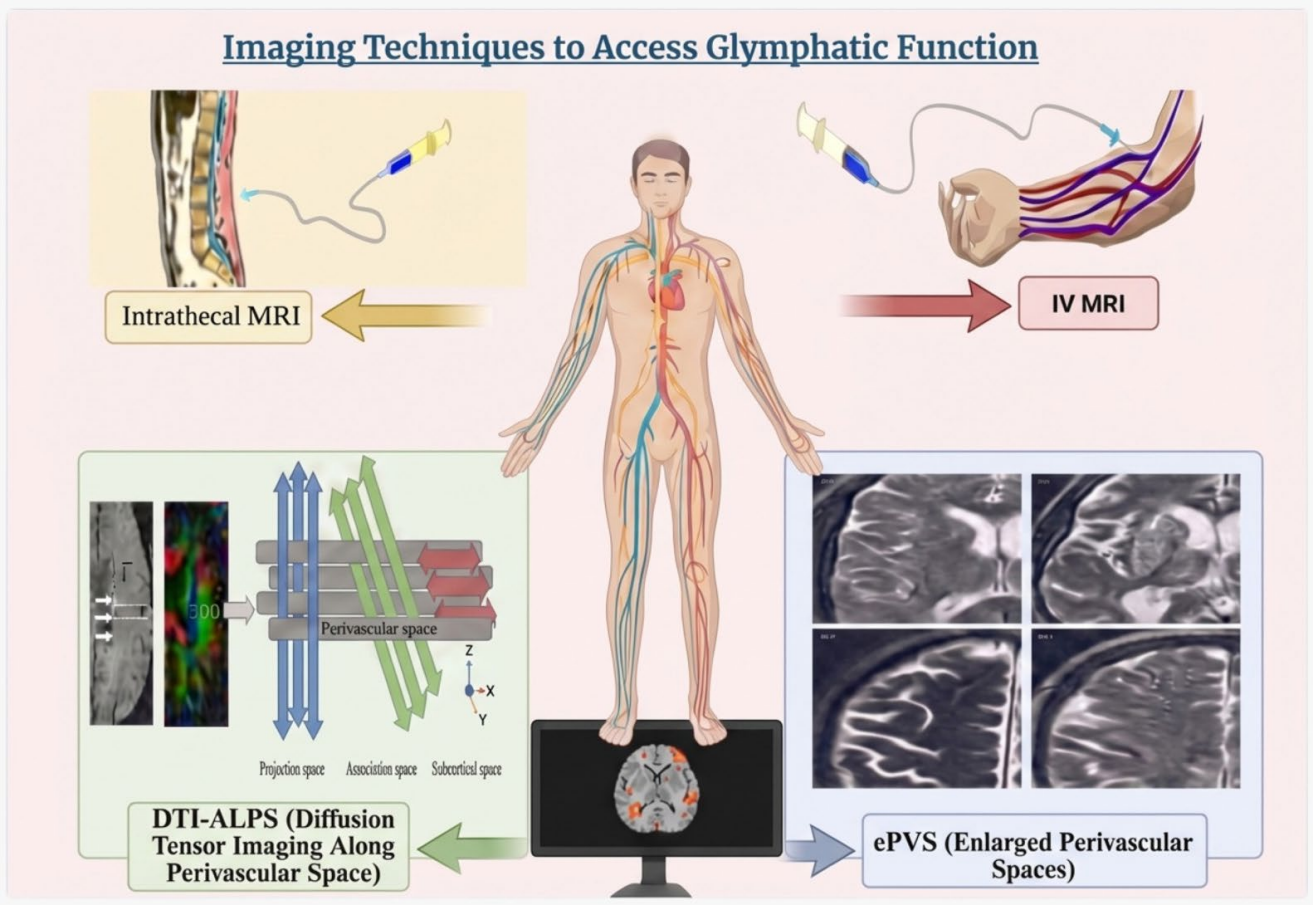
Imaging techniques to assess glymphatic function. A range of imaging modalities is used to evaluate glymphatic activity. Intrathecal and intravenous contrast‐enhanced MRI visualizes CSF flow; DTI‐ALPS quantifies directional water diffusion along perivascular spaces; and enlarged perivascular spaces (ePVS) serve as structural biomarkers of impaired clearance. Created in https://BioRender.com.

While experimental approaches, such as ultrafast MRI and optical imaging in animal models, continue to advance our mechanistic understanding, functional neuroimaging techniques, including PET and resting‐state fMRI, are also being investigated to assess the interaction between neural activity and glymphatic transport. Despite these developments, there remains a need for clinically available, non‐invasive, and standardized methods for evaluating human glymphatic function [112]. (Figure 4; Table 3).

**TABLE 3:** Imaging and experimental techniques to assess glymphatic function.

Imaging technique	Principle/Measurement	Invasiveness	Quantitative output	Clinical utility
Intrathecal Gadolinium MRI	Traces CSF flow along perivascular pathways using intrathecal gadolinium contrast	High	Time‐dependent tracer dynamics showing influx and clearance	Gold‐standard in glymphatic function in iNPH studies
Intravenous Gadolinium MRI	Uses peripheral gadolinium to infer CSF kinetics indirectly via delayed signal enhancement	Moderate	Delayed parenchymal enhancement and contrast clearance time	Feasible alternative to intrathecal route; early human studies
DTI‐ALPS	Quantifies water diffusion along perivascular spaces; indicates glymphatic efficiency	Non‐invasive	ALPS index (higher values = better flow)	Most widely used biomarker to assess glymphatic function in AD, PD, MS, and stroke
Ultrafast/4D‐MRI	Captures real‐time CSF pulsatility and flow patterns	Non‐invasive	Flow velocity maps and temporal changes	Primarily research tool; improving temporal resolution
PET/fMRI Coupling	Links glymphatic flow with neuronal activity and metabolic clearance	Non‐invasive	Regional clearance and coupling indices	Experimental translational tool in neurodegeneration

Abbreviations: CSF, Cerebrospinal Fluid; DTI‐ALPS, Diffusion Tensor Image Analysis along the Perivascular Space; fMRI, Functional MRI; MRI, Magnetic Resonance Imaging; PET, Positron Emission Tomography; PVS, Perivascular Spaces.

## 5 Therapeutic and Translational Strategies

### 5.1 Interventions Targeting Glymphatic Function in Stroke

Glymphatic flow disturbance in acute ischemic stroke is becoming increasingly acknowledged as a modifiable element of the pathophysiological cascade. Cytotoxic and vasogenic edema, which mechanically blocks perivascular spaces and hinders CSF‐ISF exchange, is often linked to the early stages of stroke. This results in the suppression of glymphatic function [12]. In addition to lowering intracranial pressure, managing edema may help partially restore glymphatic transport and accelerate waste removal during the subacute period.

Neuroprotective potential has been shown by treatments that maintain or restore AQP4 polarity after a stroke. In experimental stroke models, for instance, Yang et al. demonstrated that pharmacological suppression of MMP‐9 maintains AQP4 polarization, reduces edema, and improves outcomes [113]. Furthermore, it has been investigated whether inflammatory cascade modulators, such as dexamethasone and minocycline, might reduce the proinflammatory response and thereby promote lymphatic function [114].

Transcranial direct current stimulation and vagus nerve stimulation (VNS) are two examples of neuromodulatory treatments that are gaining popularity for their additional role in altering glymphatic flow by modulating cerebrovascular tone and autonomic regulation. There is limited data in humans, but animal research suggests that VNS may aid in the healing process after a stroke by enhancing CSF inflow and perivascular clearance [115].

Rehabilitation techniques may also play a beneficial role in restoring glymphatic activity. Regular sleep hygiene and circadian alignment may indirectly enhance brain clearance, as glymphatic transport is most active during sleep and in low‐arousal states. Cardiovascular rehabilitation and early mobility may improve cerebral perfusion and promote interstitial fluid exchange dynamics [116].

Controlling modifiable stroke risk factors, including atherosclerosis and hypertension, not only lowers the chance of recurrence but also maintains the perivascular integrity that is essential for long‐term glymphatic health [117].

Building on these mechanistic and supportive strategies, additional emerging therapies directly targeting the glymphatic system in stroke have recently been described. Experimental and translational evidence suggests that restoration of perivascular CSF–ISF exchange following ischemic injury represents a modifiable therapeutic target beyond conventional reperfusion strategies. Recent work by Yang et al. highlights that modulation of astrocytic water transport, preservation of perivascular space patency, and normalization of arterial pulsatility can significantly enhance post‐stroke glymphatic clearance [118]. These interventions were shown to attenuate cerebral edema, improve metabolic waste removal, and reduce secondary neuroinflammation, thereby contributing to improved neurological recovery. Collectively, these findings reinforce the concept that glymphatic dysfunction is not merely a secondary consequence of stroke, but an active therapeutic target whose restoration may influence both acute injury evolution and long‐term cognitive outcomes.

### 5.2 Interventions Targeting Glymphatic Function in Neurodegenerative Disease

Restoring glymphatic flow has emerged as a key treatment goal as glymphatic dysfunction leads to protein buildup in Alzheimer's disease (AD) and other neurodegenerative diseases. It has been shown that improving sleep, particularly slow‐wave sleep, greatly increases glymphatic clearance. Studies demonstrate that sleep deprivation speeds up the buildup of tau and Aβ, whereas sleep‐promoting medications such as orexin receptor antagonists such as suvorexant may provide preventive effects by boosting CSF inflow during deep sleep phases [98].

Early research is being done on pharmacological enhancers of glymphatic function. According to reports, the phosphodiesterase‐5 inhibitor tadalafil increases CSF‐interstitial exchange and meningeal lymphatic outflow, which may improve protein clearance in models of cognitive impairment [119]. Furthermore, compared to alertness and other anesthetics, the α2‐adrenergic agonist dexmedetomidine not only causes non‐REM sleep but also markedly increases glymphatic inflow [120].

Building on sleep‐based and pharmacologic strategies, recent work has further broadened the therapeutic landscape for targeting glymphatic dysfunction in Alzheimer's disease. A comprehensive analysis indicates that enhancing glymphatic clearance, through improved meningeal lymphatic drainage, cerebrovascular pulsatility, and astrocytic AQP4 polarization, can reduce amyloid‐β and tau accumulation [121]. These findings support a complementary disease‐modifying approach that emphasizes protein clearance, particularly when applied in early or preclinical stages of neurodegeneration.

In neurodegeneration, treatments must strike a balance between reducing production of damaging proteins and accelerating their clearance. Anti‐amyloid antibodies work on the former, while increasing glymphatic function works on the latter. Combining both mechanisms has the potential for more impressive outcomes.

Lifestyle modifications are also crucial. In addition to their well‐known benefits, maintaining cardiovascular health, engaging in regular physical exercise, and drinking enough water are essential for promoting glymphatic function in older adults. Perivascular integrity, a necessary pathway for CSF transport, is closely related to vascular health. Glymphatic efficiency and neurovascular unit resilience may also be supported by diets high in antioxidants and omega‐3 fatty acids [28, 120].

Glymphatic flow modulation has also shown promise in conjunction with emerging metabolic and behavioral therapies. By enhancing metabolic control and cerebrovascular function, intermittent fasting may increase autophagy and decrease the buildup of neurotoxins. On the other hand, mild to moderate alcohol use may temporarily increase CSF flow, while persistent alcohol consumption impairs perivascular clearance. It has been discovered that aerobic exercise increases interstitial solute clearance by inducing arterial pulsatility. These tactics, which include hydration and dietary modifications, may work in conjunction with pharmaceutical treatments to enhance long‐term glymphatic function and delay neurodegeneration [28].

**FIGURE 5:**
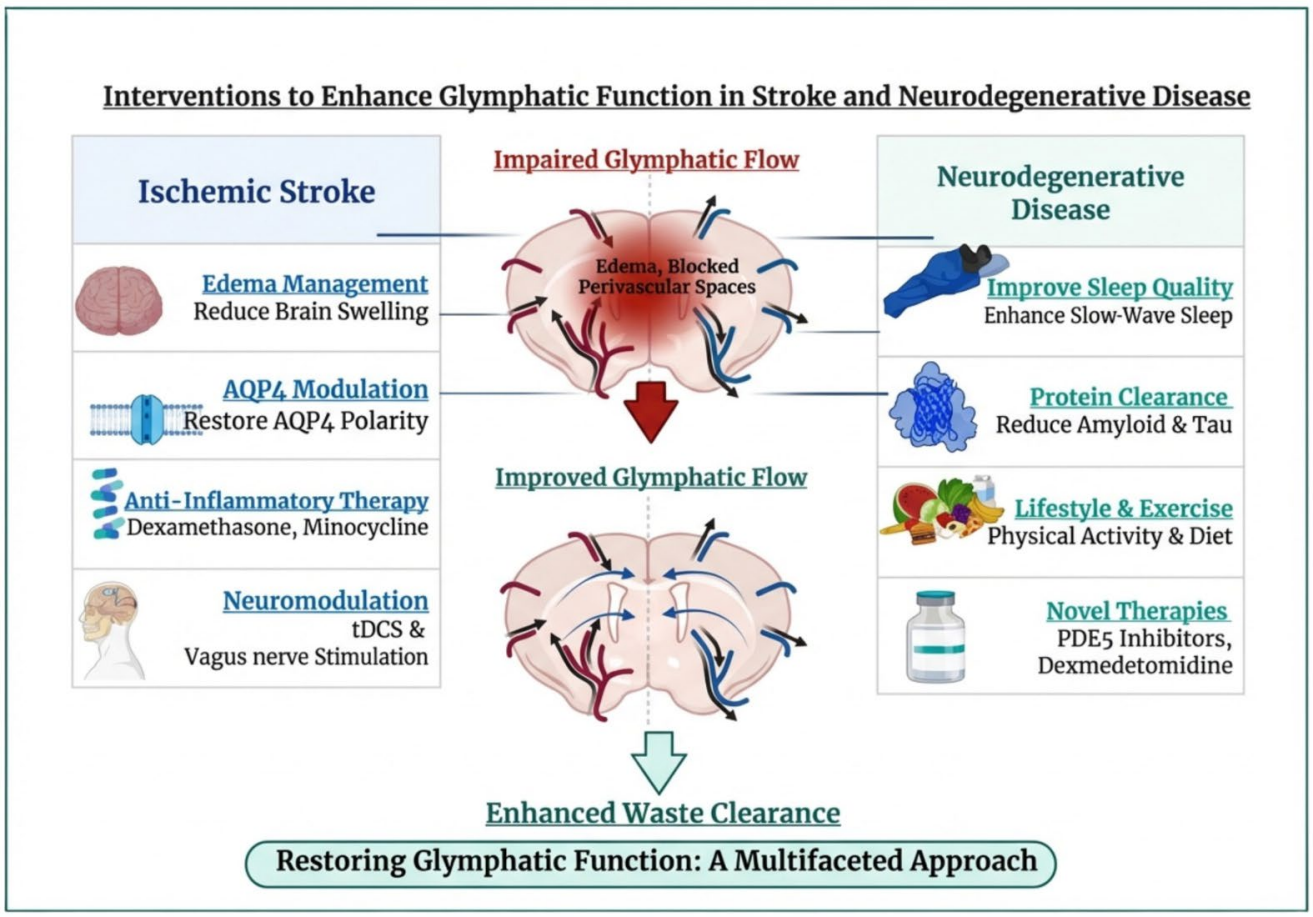
Therapeutic regimens for glymphatic dysfunctions in stroke and neurodegeneration. Overview of interventions that regulate glymphatic flux to improve CSF‐ISF flux and clearance. In ischemic stroke, the processes that affect the clearance efficiency are increased edema, inflammation, and absence of aquaporin‐4 polarization. In neurodegenerative disorders, impaired glymphatic clearance leads to accumulation of amyloid‐β and tau protein. Accordingly, in stroke, management of edema, aquaporin‐4–sparing therapeutic modalities, anti‐inflammatory therapy, and neuromodulation can revive glymphatic clearance, whereas in neurodegenerative disorders, sleep optimization, pharmacological potentiators of CSF–ISF flux, and lifestyle changes can improve clearance efficiency. The interventions above have put the regulation of glymphatic clearance in the spotlight for management and intervention in both acute and chronic conditions. Created in https://BioRender.com.

Ultimately, future research should focus on developing translational models that link glymphatic imaging biomarkers to therapeutic outcomes. It is essential to conduct clinical investigations on vascular‐targeted pharmaceuticals, sleep‐modulating medications, and glymphatic‐enhancing compounds in neurodegeneration to confirm preclinical results and inform clinical practice. (Figure 5).

## 6 Future Directions

Non‐pharmacological and non‐surgical interventions represent a particularly promising future direction for targeting glymphatic dysfunction [122]. Given the strong dependence of glymphatic activity on sleep, circadian rhythm, and autonomic regulation, strategies such as sleep optimization, circadian alignment, and treatment of sleep‐disordered breathing may offer scalable and low‐risk means of enhancing brain clearance [122]. In parallel, emerging neuromodulatory approaches, including vagus nerve stimulation, transcranial electrical stimulation, and low‐intensity focused ultrasound, have shown the ability to influence cerebrovascular dynamics and perivascular fluid flow in experimental models [123]. Lifestyle‐based measures, such as regular aerobic exercise, vascular risk factor control, hydration, and dietary optimization, may further support glymphatic efficiency by preserving arterial pulsatility and perivascular integrity [124].

There is a need for large, multicenter studies that use standardized imaging methods, such as DTI‐ALPS, dynamic contrast, and spin‐label MRI, to establish normal glymphatic flow values by age and sex. These studies would also help track how glymphatic function normally changes with time and how this differs in neurological diseases such as stroke and neurodegeneration. Early research has shown that lower ALPS scores may be linked to faster cognitive decline in Alzheimer's disease and slower recovery after surgery in iNPH. However, most studies are small and short‐term, often lasting less than a year. A longer follow‐up is essential to better understand cause‐and‐effect relationships and improve the ability to predict outcomes [125]. Furthermore, standardized integration of DTI assays into normal MRI protocol may allow for determination of baseline ALPS indices, which could serve as a valuable biomarker for the early detection or prognostic stratification of neurological disorders characterized by glymphatic failure.

Parallel efforts should concentrate on improving the accuracy, repeatability, and physiological interpretability of non‐invasive biomarkers. Combining diffusion metrics with arterial spin‐label measurements of pulsatility, high‐frame‐rate phase‐contrast MRI, and quantitative PET tracers will enable computational models to distinguish between convective and purely diffusive water transport and to relate regional flow deficits to vascular stiffness, AQP4 mispolarization, and sleep architecture. Developmental atlases that map the maturation of perivascular channels, now emerging for anterior brain regions, must be extended to posterior fossa and brain‐stem territories to support pediatric diagnostics and lifelong precision medicine [126].

Furthermore, targeting AQP4 pharmacologically remains a key research focus. In animal models of stroke, small molecules like TGN‐020 and newer compounds such as TGN‐073 have shown promise by improving AQP4 function, resulting in reduced brain swelling and enhanced waste clearance. However, before these can move to human trials, more data is needed on safety, effective dosing, and whether they can cross the blood–brain barrier. In parallel, gene‐editing and mRNA‐based strategies that increase beneficial AQP4 isoforms show potential for long‐term effects; however, challenges such as unintended actions on other brain cells and immune responses must be addressed in larger animal studies [11].

Neuromodulation offers a non‐drug approach to enhancing glymphatic function. Techniques such as vagus nerve stimulation, low‐intensity ultrasound, and 40‐Hz gamma sensory stimulation have shown promising results in experimental models, improving fluid flow and waste clearance in the brain, especially in conditions like Alzheimer's disease. However, it remains unclear whether these effects result in lasting clinical benefits. Well‐designed randomized trials, including glymphatic imaging and cognitive assessments, are needed to test their actual impact. Future studies should also investigate whether timing the stimulation, such as during deep sleep, enhances outcomes compared to when it is delivered while awake [63, 115, 123].

Future research should also consider how overall body health affects glymphatic function. Factors such as physical activity, hydration, omega‐3 intake, and the management of blood pressure and other vascular risks all influence arterial pulsations, which help drive glymphatic flow. Grouping patients based on their AQP4 or MMP‐9 genetic variants may reveal who responds best to certain lifestyle changes or treatments. In addition, combining genetics, blood flow, and imaging data through machine learning could help predict individual clearance capacity and guide personalized therapies.

Finally, future research should move beyond rodent models and include more complex systems, such as large‐brained animals and human brain organoids, to better study how drugs behave along the glymphatic pathways. These models can also help explore how the glymphatic system interacts with meningeal lymphatics and the immune system, as well as how it might be utilized to deliver treatments directly into the brain. Building open‐access data platforms and interdisciplinary research networks will be essential to improve reproducibility, set shared standards, and accelerate the development of glymphatic‐based therapies for stroke and neurodegenerative diseases [11, 125].

From a clinical‐translation perspective, sleep and circadian modulation currently appears the most feasible glymphatic‐targeted strategy because it is non‐invasive, scalable, and directly aligned with the observation that glymphatic transport is enhanced during sleep (particularly slow‐wave sleep/low noradrenergic tone) [98, 127]. Practical approaches include structured sleep optimization, treatment of comorbid sleep disorders, and pharmacologic or non‐pharmacologic enhancement of consolidated sleep (e.g., dual orexin receptor antagonists or sleep‐focused acoustic stimulation in selected populations). Key challenges include proving that sleep‐based interventions produce sustained improvements in glymphatic biomarkers (not only sleep metrics) and—most importantly—meaningful clinical endpoints, while accounting for confounders such as age, vascular stiffness, medication effects, and inter‐scanner variability in glymphatic imaging [88]. In comparison, AQP4‐targeted pharmacology remains mechanistically attractive but faces major hurdles (lack of clinically available CNS‐penetrant, cell‐selective modulators; potential context‐dependent effects on edema). Neuromodulation (e.g., VNS/ultrasound‐based approaches) is promising but requires standardization of parameters, optimization of timing (potentially sleep‐locked), and larger randomized trials with imaging‐anchored outcomes [115].

## 7 Conclusion

The glymphatic system is essential for brain health, facilitating the removal of metabolic waste and toxic proteins from the central nervous system. Disruption of this system, due to impaired AQP4 channel function, vascular changes, or poor sleep, is now linked to many neurological diseases, including stroke, Alzheimer's disease, Parkinson's disease, normal pressure hydrocephalus, traumatic brain injury, multiple sclerosis, and idiopathic intracranial hypertension. Imaging tools like DTI‐ALPS and contrast‐enhanced MRI have helped reveal how glymphatic dysfunction is associated with worse disease outcomes and cognitive decline. These insights not only offer new diagnostic markers but also highlight the glymphatic pathway as a potential treatment target. Strategies such as improving sleep, controlling vascular risk factors, and modulating AQP4 function may help restore glymphatic flow. However, more research is needed, particularly long‐term and interventional studies, to better understand how targeting this system may alter disease progression. Incorporating glymphatic insights into clinical practice represents a high‐impact diagnostic and therapeutic opportunity for a vast number of patients currently suffering from intractable neurological diseases.

## Author Contributions

**Anwar Zahran:** conceptualization; project administration; writing, original draft; writing, review and editing. **Omar Abu‐Khazneh:** literature search; data curation; writing, original draft; writing, review and editing. **Mohammad Bdair:** literature search; data curation; writing, review and editing. **Orabi Hajjeh:** literature search; writing, review and editing. **Mohammed AbuBaha:** literature search; writing, review and editing. **Waseem Shehadeh:** literature search; writing, review and editing. **Ameer Awashra:** literature search; writing, review and editing. **Ibrahim Alazizi:** literature search; writing, review and editing. **Raya Fuqha:** tables and figure preparation; visualization; writing, review and editing. **Sakeena Saife:** literature search; writing, review and editing. **Hasan Fuqha:** literature search; writing, review and editing. **Fathi Milhem:** literature search; writing, review and editing. **Husam Hamshary:** literature search; writing, review and editing. **Dana Abuzahra:** literature search; writing – review and editing. **Umar Shuaib:** senior mentorship and supervision; critical revision of the manuscript for important intellectual content; writing, review and editing.

## Funding

The authors have nothing to report.

## Disclosure

*Declaration of Generative AI Use*: No generative artificial intelligence (AI) tools or services were used in the preparation or editing of this manuscript. The authors take full responsibility for the content of this publication.

## Ethics Statement

The authors have nothing to report.

## Consent

The authors have nothing to report.

## Conflicts of Interest

The authors declare no conflicts of interest.

## Data Availability

Data sharing not applicable to this article as no datasets were generated or analysed during the current study.
